# The detection of malingering in whiplash-related injuries: a targeted literature review of the available strategies

**DOI:** 10.1007/s00414-021-02589-w

**Published:** 2021-04-08

**Authors:** Merylin Monaro, Chema Baydal Bertomeu, Francesca Zecchinato, Valentina Fietta, Giuseppe Sartori, Helios De Rosario Martínez

**Affiliations:** 1 Department of General Psychology, Università degli Studi di Padova, via Venezia 8, 35131 Padova, Italy; 2 Instituto de Biomecánica de Valencia, Universitat Politècnica de Valencia, Ed. 9C. Camino de Vera s/n, 46022 Valencia, Spain; 3 CIBER de Bioingeniería, Biomateriales Y Nanomedicina (CIBER-BBN), Zaragoza, Spain

**Keywords:** Whiplash, Malingering detection, Traffic injury, Forensic assessment

## Abstract

**Objective:**

The present review is intended to provide an up-to-date overview of the strategies available to detect malingered symptoms following whiplash. Whiplash-associated disorders (WADs) represent the most common traffic injuries, having a major impact on economic and healthcare systems worldwide. Heterogeneous symptoms that may arise following whiplash injuries are difficult to objectify and are normally determined based on self-reported complaints. These elements, together with the litigation context, make fraudulent claims particularly likely. Crucially, at present, there is no clear evidence of the instruments available to detect malingered WADs.

**Methods:**

We conducted a targeted literature review of the methodologies adopted to detect malingered WADs. Relevant studies were identified via Medline (PubMed) and Scopus databases published up to September 2020.

**Results:**

Twenty-two methodologies are included in the review, grouped into biomechanical techniques, clinical tools applied to forensic settings, and cognitive-based lie detection techniques. Strengths and weaknesses of each methodology are presented, and future directions are discussed.

**Conclusions:**

Despite the variety of techniques that have been developed to identify malingering in forensic contexts, the present work highlights the current lack of rigorous methodologies for the assessment of WADs that take into account both the heterogeneous nature of the syndrome and the possibility of malingering. We conclude that it is pivotal to promote awareness about the presence of malingering in whiplash cases and highlight the need for novel, high-quality research in this field, with the potential to contribute to the development of standardised procedures for the evaluation of WADs and the detection of malingering.

## Introduction

The term whiplash refers to the mechanism involving a sudden acceleration, followed by a deceleration, of the head-neck complex, which results in bony or soft-tissue injuries [1, 2]. Whiplash-related injuries are estimated to account for approximately 80% of all traffic injuries [2], representing the most common traffic injury and having a major impact on the economic, legal, and healthcare systems [3, 4]. Indeed, the number of insurance claims linked to whiplash are substantial, and the associated costs have increased over the past years [5], even though the rates of occurrence vary significantly across countries [2].

Whiplash injuries are characterized by the high variability of their symptoms, which may encompass diffused neck pain, neck stiffness, back pain and stiffness, headaches, fatigue, vision disorders, and dizziness. Many patients also report anxiety, depressive symptoms, memory problems, and difficulties in concentration [6]. This variety of clinical manifestations is commonly referred to as whiplash-associated disorder (WAD), a definition introduced for the first time by the Quebec Task Force in 1995 [7]. While it is generally accepted that whiplash injuries can provoke short-term symptoms, the presence of chronic whiplash symptoms, reported by approximately half of the people injured, is more controversial [8]. Notably, this is mainly due to the fact that the diagnosis of whiplash largely relies on self-reported symptoms [9]. Indeed, current medical diagnostic techniques are unable to detect soft tissue injuries accurately, which are predominant in minor WAD [1, 10]. For this reason, these disorders are difficult to diagnose and objectify and, at the same time, easy to simulate [11].

Imaging techniques (e.g., magnetic resonance imaging or computerized tomography) and physiological methods are often unable to provide useful and unequivocal information in the instances of mild injuries [12]. In the past, the suggestion was to combine various investigation methods, such as imaging techniques and psychiatric, orthopedic, and neurological data, together with a detailed clinical history and evaluation, to draw a complete diagnostic picture of a patient and a realistic level of disability [12]. However, this kind of assessment is costly in terms of time and expenses related to the instruments, it requires the presence of specialists [12], and, most importantly, does not necessarily exclude the presence of exaggerated symptoms.

Malingering (i.e., the intentional fabrication or gross exaggeration of psychological or physical conditions designed to achieve secondary benefits, such as financial compensation [13]) is fairly frequent, especially in forensic contexts and litigation evaluations, where external incentives are evident [14]. Although it is difficult to estimate the prevalence of malingering in medicolegal settings precisely, the literature indicates that it comprises 15 to 40% of cases [15–17]. As far as late whiplash-related symptoms are concerned, the literature indicates a prevalence of malingering of up to 60%, while underperformance in cognitive tests was found to be twice as frequent as in clinical contexts [18, 19]. Importantly, these percentages are likely to be an underestimate, given that successful malingerers, by definition, are not included [20]. In light of these data, it is clear that malingered WAD represents a serious economic, legal, and health issue that needs to be addressed [14].

The lack of demonstrable symptoms characterizing WAD, together with the peculiar context of the legal system, creates a situation in which policyholders may be particularly prone to exaggerate their symptomatology, motivated by the prospect of generous compensations [2, 21]. To this regard, it is relevant to consider the discrepancies in chronic whiplash-related disability and compensation seeking across Europe: in those countries where compensation rates for whiplash injuries are high, chronic whiplash is highly prevalent [22]. In Germany, for instance, WADs represent the most common consequence of road traffic accidents, counting approximately 20,000 cases each year and costing insurance companies more than 500 million euro annually [22]. Similarly, in Italy, it is estimated that the compensation for whiplash-related damages amounts to more than 2 million euro every year [3]. On the other hand, in other countries, such as Lithuania and Greece, where there is no compensation culture and no formal compensation system for late whiplash-related injuries, the development of chronic symptoms following whiplash is a rare phenomenon [21, 22]. This evidence suggests that the culture and expectations around whiplash, local insurance systems, and the prospect of monetary benefits are likely to play important roles in the prevalence of whiplash injuries and related claims, as well as in the recovery process.

The economic burden linked to WAD-related compensations makes the detection of fraudulent and exaggerated injury claims a priority. To this end, a range of biomechanical, clinical, and lie-detection techniques intended to identify malingered WADs have been proposed.

## Present paper

Regardless of the variety of methodologies, techniques, and tools developed and tested to detect malingering in medicolegal settings, there is no clear nor systematic evidence of the instruments currently available to detect malingered WADs. Given the high prevalence of WADs, substantial associated costs, and evidence suggesting that fraudulent injury claims may be particularly likely in whiplash cases, it is crucial to promote awareness about the possibility of fraudulent symptoms following whiplash as well as existing assessment tools and methods, their strengths, and their weaknesses.

The current paper aims at providing an up-to-date overview of the techniques currently available to detect malingered WAD, ranging from biomechanical and clinical measures to recently developed lie detection strategies. Advantages and limitations of each technique are highlighted and possible avenues for future research are discussed.

## Method

We conducted a targeted literature review to evaluate the current state of the art of the available methods and instruments to detect malingering following whiplash-related injuries.

### Search strategy and selection criteria

The search strategy was developed in accordance with the Preferred Reporting Items for Systematic Reviews and Meta-Analyses (PRISMA) guidelines [23]. To identify all relevant papers within the literature, the Medline (via PubMed) and Scopus databases up to September 2020 were searched, employing the following Boolean algorithm: ‘(malinger* OR faked OR feign* OR fraud* OR exaggerate* OR simulate*) AND (identif* OR detect* OR assess* OR test) AND (whiplash OR ‘neck pain’)’. No language or time restrictions were adopted in the search; however, we did exclude those papers that could not be retrieved in English at a later stage. We included all the studies that described validated measures or methods to identify malingered symptoms of whiplash-related injuries in humans. At first, in an attempt to widen our search and identify all eligible studies, we also included relevant reviews of the literature, which we then excluded at a later stage of the process.

The EndNote X9 software [24] and the Rayyan web app (https://rayyan.qcri.org) [25] were chosen to manage, screen, and review all suitable papers. At first, all titles and abstracts were screened to check whether they appeared to include techniques to detect feigned WADs. Suitable papers were then reviewed in full.

### Data extraction

Key information from the studies was recorded, including methods and tools adopted to detect malingered WAD, groups tested in the studies, whether the methods detected malingering successfully, and indexes useful to assess the quality of the measures included. We then conducted a qualitative analysis on the suitable papers and related methods and tools.

## Results

**Fig. 1:**
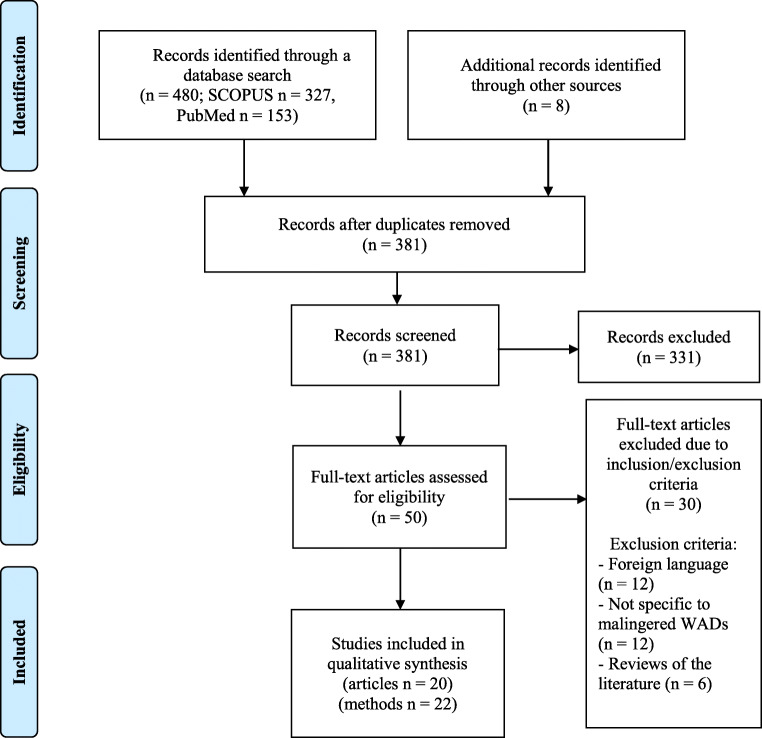
PRISMA flowchart diagram of the studies retrieved and selected for the present review

Of 382 papers screened, 51 underwent a full-text assessment. In total, 20 papers and 22 techniques adopted to detect malingered WADs (with some papers including multiple techniques) met the inclusion/exclusion criteria and were included in the review (see Fig. 1 for PRISMA flowchart).

To provide a clearer overview of the current state of the art, the methods retrieved from the papers included in the review were grouped into biomechanical techniques, clinical tools applied to forensic settings, and cognitive-based lie detection techniques (see Table 1 for a general overview).

**Table 1:** Methods and tools retrieved from the papers included in the review and grouped into biomechanical techniques, clinical tools applied to forensic settings (including the Sobel and related simulation tests, neuropsychological tools, and self-report measures), and cognitive-based lie detection techniques. The table specifies whether the tools were able to detect malingered WADs and describes the groups of participants tested in the studies. Information on the quality of the instruments, when present in the original studies, is also included

Type of Tool		Name of the Tool	Quality Assessment	Malingering Detection	Participants Tested	Authors & Year
**Biomechanical**	**Kinematics of the head-neck complex**	**CROM**	Correct identification of 87% of healthy subjects who were feigning a cervical limitation of motion	+	Healthy individuals (*n* = 25): test-retest comparisons for maximal and feigned range of motion	Dvir et al. (2001)
			N.A.	-	Patients injured in whiplash-type accidents (*n* = 25) and patients suffering from cervical degenerative changes (*n* = 25): test-retest design	Dvir et al. (2004)
		**Cervical muscle isometric strength test**	N.A.	+	Healthy control group (*n* = 18) vs. healthy subjects asked to simulate symptoms (*n* = 18), (within-subjects experimental design)	Vernon et al. (2010)
		**Fly test**	84% specificity 86% sensitivity	+	Whiplash patients’ group (*n* = 34) vs. healthy subjects asked to simulate symptoms (*n* = 31)	Gudmundsson et al. (2010)
			64.5-67.7% specificity, 79.4-82.3% sensitivity (sincere WAD vs healthy malingerers); 77.5% specificity, 79.4% sensitivity (sincere WAD vs exaggerated WAD)	+	Chronic whiplash patients’ group (*n* = 34) vs. healthy controls’ group (*n* = 31)	Oddsdóttir et al. (2015)
		**Kinematical parameters of cervical mobility**	N.A.	+	Healthy individuals (n = 20), chronic cervical pain group (n = 35), recovered WAD patients asked to reproduce a painful pattern of motion (n = 20)	Bertomeu et al. (2007)
		**Cyclical flexion–extension movement**	87% specificity 97% sensitivity	+	Healthy individuals (n = 29), Whiplash patients’ group (n = 30), recovered WAD patients asked to reproduce a painful pattern of motion (n = 30)	Baydal-Bertomeu et al. (2011)
	**Posturography**	**Stance posturography**	N.A.	+	Healthy control group (n = 20) vs. pseudo malingering group (n = 20) (within-subjects experimental design) vs. whiplash patients’ group (n = 32)	Endo et al. (2008)
		**Stance and gait posturography**	84.2-87% classification accuracy 95-100% specificity 67-78% sensitivity	+	Probable malingerers (n = 18) vs. unilateral vestibular loss patients (n = 20) vs. chronic whiplash patients’ group (n = 20) vs. healthy control group (n = 34)	Vonk et al. (2010)
	**Ocular movements and neck torsion**	**Visually guided neck movements**	N.A.	-	Whiplash patients’ group (n = 33) vs. healthy controls’ group (n = 20)	Dispenza et al. (2011)
	**Grip strength**	**Smedley dynamometer**	N.A.	+	Two cohorts of claimants undergoing medico-legal neuropsychological evaluations of head injuries. 1991-1999 cohort: moderate-severe closed head injury (n = 91), late post-concussion (n = 300); 2000-2004 cohort: moderate-severe closed head injury (n = 61), late post-concussion (n = 307)	Greiffenstein & Baker (2006)
**Clinical methods**	**Examiner evaluation**	**Sobel Test**	84,6% average inter-rater reliability	+	Patients with chronic neck pain (n = 26)	Sobel et al. (2000)
		**Simulation tests for cervical nonorganic signs**	N.A.	N.A.	Neck pain patients’ groups (n = 43 and 27) vs. healthy control group (n = 44)	Vernon et al. (2010)
	**Neuropsychological tools**	**RWRL**	N.A.	+	Two cohorts of claimants undergoing medico-legal neuropsychological evaluations of head injuries. 1991-1999 cohort: moderate-severe closed head injury (n = 91), late post-concussion (n = 300); 2000-2004 cohort: moderate-severe closed head injury (n = 61), late post-concussion (n = 307)	Greiffenstein & Baker (2006)
		**TOMM**	N.A.	+	Two cohorts of claimants undergoing medico-legal neuropsychological evaluations of head injuries. 1991-1999 cohort: moderate-severe closed head injury (n = 91), late post-concussion (n = 300); 2000-2004 cohort: moderate-severe closed head injury (n = 61), late post-concussion (n = 307)	Greiffenstein & Baker (2006)
		**ASTM**	N.A.	N.A.	Whiplash non-malingering patients (n = 65) vs. whiplash malingering patients (n = 43) vs. closed head injury controls (n = 20) vs. normal controls (n = 46)	Schmand et al. (1998)
	**Self-report**	**SCL-90-R**	N.A.	+	Whiplash patients (n = 126) vs. pain-free students asked to simulate chronic pain (n = 40)	Wallis & Bogduk (1996)
		**IPQ-R (adapted)**	N.A.	+	Student simulators: injury only (n = 33), injury plus compensation (n = 35), malingering (n = 36); clinical sample: acute (n = 27), chronic (n = 18)	Linnell & Easton (2006)
		**MMPI-F**	N.A.	-	Two cohorts of claimants undergoing medico-legal neuropsychological evaluations of head injuries. 1991-1999 cohort: moderate-severe closed head injury (n = 91), late post-concussion (n = 300); 2000-2004 cohort: moderate-severe closed head injury (n = 61), late post-concussion (n = 307)	Greiffenstein & Baker (2006)
		**MMPI-CCS**	63% classification accuracy 94% specificity 30% sensitivity	+	Litigants who passed previous symptom validity tests (n = 50) vs. those who did not pass them (n = 50) vs. non litigants’ group (n = 50)	Henry et al. (2014)
		**SRSI**	100% specificity 90% sensitivity	+	Honest control group (n = 40) vs. subjects asked to simulate symptoms (n = 40) (within-subjects experimental design)	Giger & Merten (2019)
		**SIMS**	100% specificity 80% sensitivity	+	Honest control group (n = 40) vs. subjects asked to simulate symptoms (n = 40) (within-subjects experimental design)	Giger & Merten (2019)
		**Whiplash syndrome questionnaire**	Correct identification of 94% of simulators and 84% of the exaggerators	+	Subjects with whiplash syndrome asked not to simulate (n = 25) vs. subjects with whiplash syndrome asked to simulate/exaggerate symptoms (n = 25) vs. controls asked to simulate symptoms (n = 25) vs. controls without instructions (n = 25)*	Sartori et al. (2003)
**Cognitive-based lie detection techniques**		**aIAT**	90% accuracy to in detecting malingering of whiplash-related injuries	+	N.A.	Sartori & Agosta (2007)

*Note.* + = significant difference between the groups compared (i.e., the technique is effective in discriminating malingerers from genuine participants); - = non-significant difference between the groups compared (i.e., the technique is not effective in discriminating malingerers from genuine participants); *N.A*. information not available or not measured. *CROM* cervical range of motion; *RWRL* Rey Word Recognition List; *TOMM* Test of Memory Malingering; *ASTM* Amsterdam Short Term Memory; *SCL-90-R* Symptom Checklist-90-Revised; *IPQ-R* Illness Perception Questionnaire-Revised; *MMPI-F* Minnesota Multiphasic Personality Inventory-Infrequency Scale; *CCS-MMPI* Cognitive Complaints Scale from the MMPI-2; *SRSI* Self-Report Symptom Inventory; *SIMS* Structured Inventory of Malingered Symptomatology; *aIAT* Autobiographical Implicit Association Test. *In a cross-validation study, 40 additional participants (10 per group with the same clinical and demographic characteristics as the first groups) were recruited [26]

### Biomechanical techniques

To provide objectivity to the clinical assessment of whiplash-related injuries, some authors have proposed considering biomechanical parameters, such as the kinematics of the head and neck, posture, eye movements, and grip strength. Broadly, these methods are based on the evaluation of movements performed multiple times and/or under different circumstances, a strategy that helps reveal inconsistencies between repeated performances or abnormal and improbable patterns of impairment.

#### Kinematics of the head-neck complex

##### Performance of restriction of cervical range of motion (CROM)

This strategy is focused on the comparison between maximal (i.e., genuine) and submaximal (i.e., feigned) cervical motions [27, 28], based on the rationale that malingerers are not able to accurately repeat their feigned head movement disturbances multiple times. Dvir et al. [27] demonstrated that the coefficient of variation (CV)—a consistency index related to a sequence of repetitive movements—represented an accurate, efficient tool to detect submaximal effort in performing cervical movements among healthy subjects. At first, participants were instructed to move the head in all the primary directions (i.e., flexion, extension, right and left rotation, and right and left lateral flexion). Next, they were asked to repeat the same movements, but pretending that they had experienced a neck injury and were feeling pain. The same protocol in reverse order was repeated a few weeks later. Importantly, the range of motion-based CV allowed researchers to correctly identify 87% of those who were feigning a cervical limitation of motion. The same procedure tested in a second study [28] among whiplash patients and patients suffering from cervical degenerative changes (CDC) did not produce the same encouraging results. The findings showed that the submaximal effort (i.e., movements performed as if suffering from an intense pain) was significantly and consistently associated with a compression of cervical motion and with higher variation of performance. However, the CV was not effective in differentiating between maximal and submaximal effort for patients injured in whiplash accidents or CDC patients. Nevertheless, a logistic model allowed the identification of a coefficient of variation-based cutoff that may aid in the recognition of intentional exaggeration of motion restriction, using pain as a cue. Importantly, this model is sensitive to sample size, and further studies based on much larger samples are warranted. Given that the CV was able to discriminate between maximal and submaximal efforts among normal subjects [27] effectively but not among whiplash or CDC patients [28], future investigations and replications are needed.

##### Cervical muscle isometric strength test

The measure of isometric flexion, extension, and bilateral bending contractions of the head/neck muscles is another proposed marker of insincere effort among neck pain patients. In a study by Vernon et al. (2010) [29], healthy participants underwent cervical muscle isometric strength tests in two conditions: up to comfortable maximum and during simulated malingering [29]. The signs of malingering in the submaximal effort were found to be i) consistently reduced maximum strength values ​​in all measurement ranges without gender differences; ii) greater inconsistency within trials, probably due to volitional inconsistency; iii) lack of replication of the expected performance norms. Specifically, in simulated malingering, the typical gender differences disappeared, and the normative flexion/extension ratios were not only statistically insignificant, but also higher than those of actual whiplash patients and neck pain patients. These findings could be explained by the fact that malingerers were unaware of the ratio between flexion and extension of neck muscles’ values and by the evidence that in patients with whiplash-type injuries, the flexor muscles weaken more than the extensors [29]. Future replications of this study, testing larger samples and including patients instead of healthy students, are desirable; moreover, it would be beneficial to combine this strategy with techniques targeting other symptom dimensions.

##### The Fly Test

The Fly Test [10, 30] is a computerised method developed to measure patients’ cervical spine movements (i.e., head and neck movements) on a moment-to-moment basis [31]. The test asks participants to follow the fly (i.e., target cursor on the computer screen), moving their head and completing tasks of various difficulty levels. Head movements are registered by sensors placed on the subjects’ heads [31, 32]. This strategy was used to accurately differentiate patients with real WAD from fakers who deliberately simulated symptoms and from patients who exaggerated their symptoms [31]. Gudmundsson et al. [31] tracked and recorded head movements using the Fly Test, computed the difference between the measured and actual trajectories, and used the data as input to support vector machine classifiers. The authors stated that their study represented the first example of machine learning algorithm applications in the field of malingered WAD identification. Their findings were encouraging, achieving 86% sensitivity and 84% specificity from an ensemble classifier based on a cross validation method, with the medium difficulty level being the more informative task among the three examined. Hence, the Fly Test coupled with artificial intelligence models may represent a valid, non-invasive, and cost-effective method to detect fraudulent claims. Through a cross-sectional study, Oddsdottir et al. (2015) adopted the Fly Test to explore whether it could discriminate between WAD patients, healthy subjects deliberately faking WAD symptoms, and WAD patients (persistent WAD grade II [7]) exaggerating their condition [32]. The authors correctly classified 71.8–81.5% of the participants. Two parameters utilized in this study accurately differentiated the feigned performance of the asymptomatic group from the WAD group’s sincere effort (amplitude accuracy cut-off: 79.4% sensitivity and 67.7% specificity; time on target cut-off: 82.3% sensitivity and 64.5% specificity). All the three parameters tested (amplitude accuracy, time on target, and jerk index) could differentiate genuine from feigned performances in the WAD group (sensitivity = 79.4% and specificity = 77.5%) [32]. Despite the promising results of this tool, it is argued that it cannot be applied in the evaluation of severe WAD patients.

##### Motion patterns and parameters

The kinematical measurements obtained from cervical and neck mobility allow the determination of parameters used in detecting malingering among neck pain patients [33, 34]. Specifically, the analysis of kinematical parameters (i.e., range of motion, angular velocity, angular acceleration, and harmonicity) while completing flexo-extension, lateral bending, and rotation movements was shown to accurately detect simulators of cervical pain, distinguishing them from genuine patients [33]. Baydal-Bertomeu et al. (2011) [34] developed a protocol consisting of a biomechanical assessment system for the cervical spine that generates objective information through the kinematic analysis of the cervical movements and uses a non-invasive technique based on inertial portable sensors. The authors argue that this protocol allows clinicians to quantify the degree of pathology and its limitations in patients’ movements, keeping the evolution and rehabilitation activities under control and thus helping the identification of simulation. The system’s instrumentation consists of two inertial sensors, one placed on the forehead and one placed on the upper part of the participant's back (seventh cervical vertebra). Subjects are asked to make three cyclical movements (flexion extension, lateral flexion, and axial rotation) for 30 s at a comfortable speed and reaching the maximum motion of the cervical joint (limit test); each movement is repeated twice. Participants also carry out a functional test, which consists of observing three target stimuli (numbers) that appear on the screen lamps placed above their head and transcribing them on a whiteboard. Once all the tests have been carried out, the system generates three global indexes—a mobility index, speed index, and collaboration pattern index—allowing simultaneous analysis of the repeatability and consistency of the results with the rest of the evaluations and detection of non-collaborative or simulation behaviours. These studies suggest that motion patterns and parameters may represent informative and valid methods to identify suspect claimants and abnormal patterns of movement and should be used to complement other diagnostic tools, enhancing the accuracy of evaluations and the confidence of diagnoses [33, 34].

#### Posturography

The techniques included in this section are focused on the analysis of patients’ balance while standing in a static upright position as well as in dynamic conditions. These strategies appear particularly useful in whiplash patients presenting with vertigo and dizziness and allow examination of the vestibular and proprioceptive systems’ influences on postural control and balance [35].

##### Stance posturography

One of the proposed methods to detect malingered whiplash symptoms involves the recording of static posturography parameters and the analysis of upright posture with open and closed eyes. In a cross-sectional study, Endo et al. (2008) [36] investigated typical symptoms of whiplash patients ), such as vertigo and dizziness, as well as how these symptoms could be faked by those attempting to simulate this pathology. Observations of healthy malingerers included differences from real patients in a number of specific parameters, such as a wider total envelope area (the area within the outer shift line) in a unit of time, a long sway length per second, and a low Romberg rate (representing eyes-closed/eyes-open in length per second) [36].

##### Stance and gait posturography

Additional gait and stance tasks were tested to differentiate malingerers from genuine whiplash patients. In their study, Vonk et al. (2010) compared four groups (probable malingers, unilateral vestibular loss patients, chronic whiplash patients, and healthy subjects) in fourteen gait and stance tasks (20 s long) of increasing difficulty [35]. In gait tasks, malingerers presented a larger extent of trunk sway, while in stance tasks, they showed greater sway in both roll and pitch directions, compared to real patients’ groups and healthy subjects. Regarding the accuracy in participant discrimination, 84.2% of participants were correctly classified as malingerers or genuine whiplash patients, with 100% of patients and 67% of malingerers correctly identified. Instead, using the eleven criteria developed by the authors based on the inconsistent performance principle, the authors correctly discriminated between malingerers and whiplash patients with 87% accuracy, with 78% of malingerers identified and 95% of real patients. While posturography techniques represent brief, simple, and non-invasive strategies that appear to effectively distinguish malingerers*,* analysis of posture alone cannot establish the physical site that is causing the balance dysfunction and does not provide a complete picture of the symptom presentations; hence, it needs to be combined with other measures [35, 36]*.* Furthermore, more research is necessary to compare patients exaggerating their symptoms and those instructed to act as malingerers [35, 36].

#### Ocular movements and neck torsion

Evidence from the literature suggests that the smooth pursuit neck torsion test (SPNT) can be used to distinguish patients with whiplash from other diagnoses of non-traumatic neck pain as well as to accurately differentiate between whiplash patients with and without dizziness from healthy controls [37–39]. Tjell et al. (2002) stated that the SPNT would be difficult to fake consistently, given that malingered eye movements tend to be rather bizarre saccades of varying dimensions, making them easy to identify [38]. However, Dispenza et al. (2011) found discrepant results. Specifically, they focused on visually guided eye movements and compared a control group and a group of whiplash patients presenting balance deficits following their injuries [40]. They found no significant differences in the two groups’ saccadic and smooth-pursuit movements. They based their study on the evidence that, because of the whiplash injury, it is plausible to identify lesions in various cervical sites with subsequent weakness in the nervous system. Consequently, evaluation after trauma includes assessments of the peripheral labyrinth and vestibulo-ocular reflex system as well as evaluation of visually guided movements of the eyes through video-oculography/video-nystagmography. The authors concluded that for patients without lesions of the cerebral spine, which constitute the majority of common whiplash cases, ocular motor alterations are improbable and post-whiplash alterations of saccadic and smooth-pursuit movements, as reported in previous literature, are probably due to impairments in attentional processing after trauma [41]. Thus, the authors suggest that the analysis of ocular movements cannot effectively differentiate a genuine patient from a malingerer following whiplash injuries [40].

#### Grip strength

The Smedley dynamometer, an instrument commonly used to measure grip strength [39], was proven accurate in detecting atypical post-concussion presentations [40] and represents another method used to assess simulation of whiplash-related damage. In their study, Greiffenstein and Baker (2006) [42] adopted the Smedley dynamometer to detect malingering of motor symptoms among a group of compensation seekers and found that patients presenting chronic whiplash and minor head-injury were more likely to show invalid signs and exaggerated weakness, compared to severely injured persons, on measures of simulated motor deficits. Strikingly, more severe compensable injuries were associated with less weakness than minor injuries. However, this tool was used in combination with other instruments and measures, so it is not possible to draw any definite conclusion on the efficacy of the Smedley dynamometer alone in detecting malingered motor symptoms following whiplash.

### Clinical tools and criteria applied to forensic practice

One of the classical approaches to malingering detection is based on the application to forensic contexts of the qualitative observation of symptoms and behaviours, adopting clinical and epidemiological principles.

In terms of manifestations of symptoms, some of the most popular and validated detection strategies are based on the identification of either *unlikely* presentations of symptoms or *amplified* symptoms [43]. The former category includes the observation of rare symptoms, which relies on the evidence that malingerers tend to overreport symptoms that occur very infrequently among genuine clinical patients or non-clinical populations [44, 45]. Similarly, the strategy focused on the detection of improbable symptoms is based on the observation of the endorsement of extreme, abnormal, and preposterous symptom patterns among malingerers [44, 45]. Assessment of symptom combinations is another strategy to identify psychiatric malingering, and it focuses on unusual symptom pairs that are common alone but rarely seen together in genuine patients [44, 45]. Strategies of *amplified* presentation are based on findings indicating that, compared to genuine patients, malingerers are likely to refer a larger number of symptoms (indiscriminate symptom endorsement strategy), are more prone to describe their symptoms as ‘extreme’ or ‘unbearable’ (symptom severity strategy), tend to present symptoms that are easily linked to serious disorders (obvious symptoms strategy), and are likely to over-endorse common misconceptions and stereotypes that individuals have towards specific syndromes (erroneous stereotypes strategy) [44, 45]. Moreover, it is often useful to compare individuals’ reports of their symptoms to more objective clinical observations, which often reveals discrepancies among malingerers (reported vs. observed symptoms) [45]. These rules can be applied when conducting clinical evaluations; however, they also led to the development of instruments to identify malingering, especially in the field of mental illness, such as the Structured Interview of Reported Symptoms (SIRS) [46] and the Miller Forensic Assessment of Symptoms (M-FAST) [47]. Common general strategies for detecting malingerers in cognitive impairment consist of identifying patterns of cognitive symptoms that are either *excessive* (e.g., performance failures on simple tasks that normally can be completed even by severely impaired patients) or *unexpected*. (e.g., identification of failure patterns that are statistically unlikely) [44, 45]. For instance, the *floor effect* strategy seeks to identify malingerers by presenting tasks that are too simple to fail but that malingerers may not recognise as simple, whereas the *symptom validity testing* (SVT) strategy identifies malingerers based in their performance on forced-choice tests [44]. Overall, these strategies and criteria can guide clinical evaluations intended to detect feigned symptom presentations, but they are also behind the development of measures and instruments able to identify suspected symptoms, which are commonly used in clinical assessments or specifically designed for forensic practice [45, 48, 49].

Below, the clinical, neuropsychological, and self-reported measures that researchers have adopted to identify malingering, specifically in the context of whiplash injuries, are presented.

#### Sobel test and related simulation tests

The Sobel test is a tool designed to screen for non-organic cervical symptoms and signs. Researchers began to show interest in non-organic symptoms and signs in the early twentieth century, motivated by the idea that they indicated some sort of simulation [50]. Indeed, in 1954, Brown et al. devised a list of criteria for identifying the psychogenic cause of low back pain [51], and subsequently, Waddell et al. (1980) developed and demonstrated the reliability of a group of eight lower-lumbar physical signs of non-organic back pain [52]. The tool designed by Sobel (2000) was based on Waddell's test of nonorganic physical signs of low back pain; signs conforming to the cervical spine were derived from Waddell’s test, and three ad hoc signs for non-organic cervical symptoms were added. The Sobel screening tool [50] must be administered by two clinicians (84.6% average inter-rater reliability) and consists of the evaluation of a group of non-organic cervical signs that signal the presence of abnormal illness behaviour among patients manifesting neck pain. This technique includes the examination of eight physical signs, grouped into five categories: 1. *Tenderness*: superficial and nonanatomic; 2. *Simulation*: head/shoulder/trunk rotation in sitting and standing positions; 3. *Range of motion*; 4. *Regional disturbance*: sensory loss and motor loss; 5. *Overreaction*. Simulation is suspected when positive results are obtained on two or more physical signs. In the literature, there are also criticisms of the measurement of non-organic signs as a malingering detection tool. Fishbain et al. (2003) argue that Wandell’s signs are not associated with secondary gain [53]. Mendelson and Mendelson suggest that non-organic signs are not synonyms of malingering (especially in terms of low back pain) and that other evaluation methodologies are necessary to make this determination, such as facial expression evaluation, mechanical testing, differential spinal block implementation, thermography amytal evaluation, and pentothal administration [54]. Overall, the Sobel test represents a standardised and valid tool for assessing cervical non-organic signs that may be indicative of abnormal illness behaviour (i.e., malingering). However, the assessment of WAD requires caution and a careful evaluation of a variety of symptoms.

Other researchers specifically revised the simulation signs presented in the Sobel test. Vernon et al. (2010) [55] developed four simulation tests for cervical nonorganic signs and tested them among groups of patients suffering from neck pain and a control group: seated trunk rotation (with manual neck contact by the examiner), cervical ranges of motion (with at least 10% difference between active and passive movement), palpation of the mastoid process (Libman's test, not applicable for subjects experiencing upper cervical spine pain), and side-lying passive shoulder abduction (with manual neck contact by the examiner) [55]. The logic behind these simulation tests of nonorganic signs is that healthy subjects not experiencing pain and neck pain (i.e., sincere patients) should not feel any pain while engaging in these specific manoeuvres, but at the same time, the movements are perceived as appropriate for a neck examination (i.e., good face validity). If a person shows any pain reactions, it is likely their symptoms are non-organic or may be simulated. However, these simulation tests need further investigations in terms of reliability (especially among real patients instead of chiropractic students) and their potential for use as indicators of nonorganic pain in the context of the fear avoidance model in neck pain assessment [55].

#### Neuropsychological tools

The following neuropsychological tools were designed to screen for the simulation of cognitive deficits but have also been tested specifically in the context of whiplash-related injuries and suspected malingering.

##### Rey Word Recognition List (RWRL)

The RWRL is a simple verbal recognition measure that was originally developed to detect noncredible cognitive performances and has been widely used to assess malingering of memory deficits [56, 57]. This recognition task is based on a floor effect strategy [44]. Hence, the presence of significant mistakes is suspicious. Greiffenstein and Baker (2006) [42] used this test to measure the simulation of memory complaints among compensation seekers and found that patients presenting chronic whiplash and minor head injuries were more likely to show poor memory performance, compared to severely injured persons. However, the RWRL was used in combination with other measures, and this prevents us from drawing any definite conclusion on its ability to detect malingered memory symptoms following whiplash. Moreover, although the RWRL is a brief and cost-effective instrument, it is not clear whether it has adequate specificity and sensitivity [45, 57]. Importantly, the test should be validated specifically to detect malingered symptoms following whiplash.

##### Test of memory malingering (TOMM)

The TOMM [58] is a forced-choice recognition memory test based on SVT and a *floor effect* detection strategy (i.e., scores significantly below 50% are indicative of malingering) [45]. Greiffenstein and Baker (2006) [42] used this test to screen for memory loss simulation among a cohort of insurance claimants with head injuries. The authors found that patients presenting chronic whiplash and minor head injuries were more likely to show poor memory performance, compared to severely injured persons, indicating possible or probable simulation. However, as different tools were used together to detect various aspects of simulation, further research is warranted to test and validate the TOMM specifically for malingering in whiplash cases [59].

##### The Amsterdam Short-Term Memory (ASTM) Test

The ASTM test [60] is an instrument developed specifically to detect cognitive malingering. It is based on an SVT paradigm and consists of a short memory test alternating with distractors (i.e., simple mathematical calculations). The validation study [60] showed that the test could accurately discriminate between genuine patients with closed head injuries and healthy subjects who had been asked to fake memory disturbances. Given its high sensitivity to suboptimal memory performance, Schmand et al. (1998) [18] adopted the ASTM test as a screening tool for malingering in a group of post-whiplash patients [60]. The authors estimated a prevalence of underperformance of 0.61 in patients involved in litigation and of 0.29 in non-litigation patients. Next, a variety of conventional neuropsychological tests were administered. The results suggested that malingering post-whiplash patients scored as poorly as patients with closed head injuries, and the authors conclude that the extremely poor performance of some post-whiplash patients must be explained by under-performance rather than genuine organic brain dysfunctions [18]. Importantly, this study did not validate the use of the ASTM test as a screening tool specifically in the context of whiplash injuries. Furthermore, no sensitivity or specificity indexes were reported; thus, no definitive conclusions can be drawn.

##### Self-report measures

The self-administered measures presented below include two types of tests—stand-alone tests and scales embedded in questionnaires used for a variety of purposes—that have been adopted to assess malingered WAD.

###### Symptom Checklist-90-Revised (SCL-90-R)

The SCL-90-R [61] is a tool commonly used for clinical evaluations that consists of a self-reported checklist of 90 items. Items describe a range of physical and psychological symptoms referring to somatization, obsessive-compulsive, interpersonal sensitivity, depression, anxiety, hostility, phobic anxiety, paranoid ideation, and psychoticism; respondents are required to indicate on a 5-point scale how much they were bothered by each symptom over the previous week.

Wallis and Bogduk (1996) [62] assessed the validity of the SCL-90-R as a screen for possible malingering in whiplash-related pain. In their experiment, researchers asked healthy students to feign chronic pain following a road traffic accident to gain monetary compensation and compared their response patterns to the checklist of responses from by whiplash patients. Their results yielded a significant difference between the two groups. Specifically, the students scored significantly higher than patients on all subscales (*p*s < .001), suggesting that it is difficult to fake a psychological profile in the SCL-90-R typical of a whiplash patient. Hence, this questionnaire can be considered a robust measure against deliberate simulation. In particular, the findings showed that those simulating whiplash injuries tend to overestimate the severity of the pain, scoring high across all the subscales of the questionnaire. On the contrary, whiplash patients exhibit a characteristic profile with modest elevations only on the somatization, obsessive-compulsive, and depression subscales, a pattern that the students were not able to replicate. However, as noted by Dvir et al. (2004) [28], the study lacks specificity and sensitivity indexes; hence, it is not possible to estimate cut-off values or, consequently, to determine the sincerity of claims from psychological symptoms of whiplash injuries.

###### Illness Perception Questionnaire-Revised (IPQ-R)

Linnell and Easton [63] focused on illness perceptions and perceived severity of pain to analyse the effect of compensation in the context of simulated WADs. To this aim, the authors used an adapted version of the IPQ-R [64] and compared beliefs among three simulated conditions (one without compensation, so that genuine beliefs about whiplash injury could be measured, one with compensation, and one simulating malingering) to those of a clinical sample of actual litigant claimants (divided into acute and chronic stages). The study revealed that those who have been suffering from whiplash-related symptoms for some time tend to hold negative beliefs about the consequences of their condition that are more extreme than those explicitly asked to malinger the same condition. The authors suggest that beliefs about whiplash injury may represent possible indicators of malingering behavior; however, further studies are warranted to test this hypothesis.

###### Minnesota Multiphasic Personality Inventory-2 (MMPI-2)

The MMPI-2 [65] is a 567-item self-report instrument originally designed to assess personality and psychopathology [66] that has been used extensively outside of clinical settings. It includes validity indicators as well as consistency scales that make it particularly suited for forensic applications [67]. In order to assess the presence of psychiatric symptoms simulation among compensation seekers following head injuries, Greiffenstein and Baker (2006) [42] adopted the infrequency scale of the MMPI-2 (MMPI-F), which provides information on the presence of symptom presentations that are relatively uncommon in the general population. Specifically, high scores on the F scale are generally an index of symptom exaggeration [42]. The authors found that the mean MMPI-F score did not reach the critical score of 100 T necessary to invalidate an MMPI profile [68] and that there were no between- or within-group differences for this scale, reinforcing the evidence that the MMPI-F scale is not sensitive enough to detect psychiatric malingering among personal injury litigants [42, 69]. Henry et al. (2014) [70] describe the development of the 13-item Cognitive Complaints Scale (CCS) embedded in the MMPI-2, with most of the items not belonging to the F validity scales. This subscale can be used in forensic settings to assess the validity of self-reported cognitive symptoms. The findings indicate that the CCS has a high specificity (94%) and a moderate classification accuracy (63%) when a cut-off score of 12 or more is adopted for distinguishing the group of subjects that did not pass SVTs, such as the TOMM [58], the Word Memory Test (WMT) [71], and the Victoria Symptom Validity Test (VSVT) [49], from the group that did pass them successfully. Specifically, the former group scored significantly higher on the CCS compared to the latter. It should be noted that the two groups considered consisted of litigants with mild head injuries and disability claimants, therefore with known external incentives. Among them, 8% of the participants in first group and 12% of the second presented whiplash-related injuries. The authors report the low sensitivity of the scale (30%) as a limit [70]. However, they argue that this scale may represent a helpful aid in the discrimination of malingerers, especially when performance tests are not available (freestanding and cognitive test measures).

###### Self-Report Symptom Inventory (SRSI) and Structured Inventory of Malingered Symptomatology (SIMS)

In their article validating the French version of the SRSI [72], a stand-alone self-report questionnaire made up of 107 items to test symptom validity, credibility, and overreporting, Giger and Merten (2019) [73] asked healthy subjects to fill out the SRSI and the SIMS [74], first in an honest condition, and then faking symptoms after a whiplash accident. The SIMS is a self-report questionnaire conceived to detect malingering of psychiatric symptoms as well as symptoms of cognitive impairment [74]. The authors criticized the SIMS, claiming that, being composed only of items of pseudosymptoms, it does not have a sufficient appearance of face validity, making it easily identifiable as a test for the detection of malingered symptoms. In contrast, the SRSI includes genuine and pseudosymptom scales to conceal the real aim of the test. Furthermore, the related score reliabilities of the genuine and pseudosymptom scaled were high, with Cronbach’s alphas of .95 and .92, respectively. With a standard cut-off (> 9, 5% of maximum false-positive rate), researchers obtained a specificity of 100% (honest condition) and a sensitivity of 90% (malingering condition) in the classification using the SRSI and specificity of 100% (honest condition) and sensitivity of 80% (malingering condition) using the SIMS (cut-off >16). Finally, the SIMS scores and the SRSI number of reported pseudosymptoms had a 0.69 correlation. It should be noted that the majority of those asked to simulate their symptoms faked their answers concerning cognitive problems such as memory and concentration impairments or simulated pain.

###### The Whiplash Syndrome Questionnaire (WSQ)

The WSQ [26] developed by Sartori et al. is a brief self-report measure that has been shown to screen for feigned symptoms accurately. The questionnaire includes eight scenarios, each with ten response options (e.g., *making an important group decision* or *spending a long time writing*) that responders ranked according to the ease with which each given action can be performed [26]. The assumption behind this measure is that only those suffering from authentic injuries are able to discriminate between easily doable vs. difficult daily actions or movements accurately. In a small validation sample, the questionnaire was shown to correctly identify 94% of the simulators and 84% of the exaggerators [26]; moreover, the proportion of false positives was fairly low [26], suggesting that the WSQ may represent a promising, reliable, and advantageous tool to screen for feigned WADs. However, it does not provide a complete and exhaustive assessment; hence, we recommend adopting it in combination with additional measures.

### Cognitive-based lie detection techniques

Cognitive-based lie detection measures rely on the cognitive load or the limited resources that the cognitive system has available when performing mental tasks [75]. Literature has shown that lying is more cognitively demanding than truth-telling [76]; hence, deception can be reflected, for instance, in longer response times, greater hesitancy, and higher numbers of errors [45]. Cognitive load-inducing lie detection techniques are aimed at enhancing respondents’ cognitive efforts during tasks, for instance, via complex sentences [77] or unexpected questions [78], making it significantly harder to lie than to respond honestly. Recently, a range of cognitive-based lie detection techniques has been proposed. These techniques focus on the analysis of implicit behaviours, such as mouse movements [79], keystroke patterns [80], and eye movements during interviews [81]. However, to our knowledge, only the Autobiographical Implicit Association Test (aIAT) [82] has been specifically applied to WAD malingering detection.

#### The Autobiographical Implicit Association Test (aIAT)

The aIAT [82] is a computer-based method that builds on the evidence of the cognitive mechanisms involved in lying. Specifically, the instrument detects lies by recording individuals’ response times (RTs) during classification tasks. The increased cognitive effort required for lying, as compared to telling the truth, leads to slower reaction times when responding to questions (e.g., related to whiplash symptoms). Because it relies on implicit indexes rather than conscious behavior (e.g., self-report measures), this method is particularly suited for forensic settings, where simulation is likely. In a preliminary study, the aIAT successfully detected the malingering of whiplash-related injuries, showing an overall accuracy of approximately 90% [83]. However, this is just a preliminary study that needs to be expanded before definitive conclusions can be drawn.

## Discussion

Insurance claims related to injuries following whiplash-related accidents are substantial, associated with high costs, and have a significant impact on healthcare, legal, and economic systems worldwide [3, 4]. Possible symptoms related to whiplash are numerous and heterogeneous, and the absence of demonstrable pathoanatomical signs that characterise chronic WAD makes its diagnosis largely based on self-reported symptoms, hence, particularly vulnerable to malingering [11]. Indeed, the need for accurate assessment methods that take into account the wide range of symptom manifestations was recognised long ago [12], and suspicious symptom presentations among whiplash claimants represent a well-known and longstanding problem [2]. However, these issues have not been systematically addressed.

Originally, to deal with the variety of symptoms associated with whiplash injuries, researchers suggested including various professional figures and scientific methods in the evaluation. However, this approach comes with considerable economic and time costs, and, importantly, no guarantee of malingering identification [12]. Over the years, numerous strategies to provide more objective evaluations for detecting feigned presentations have been proposed.

The present literature review identified 22 strategies, including biomechanical methods, clinical measures, and lie detection techniques, developed for the detection of malingered WADs or adapted to that purpose.

Among the biomechanical tools, four macro-areas of investigation were identified, namely head and neck movements, posture, eye movements, and grip strength. Broadly speaking, these techniques are intended to detect malingering through the identification of inconsistent performances, inability to accurately replicate motor impairments, and exaggerated deficits. The Fly Test [31, 32] showed promising results, as did the analysis of motion patterns and parameters [33, 34] and posturography techniques [35] for recording real-time movements. Interesting future avenues could be derived from the integration of these strategies with artificial intelligence algorithms, as proposed by Gudmundsson et al. [31]. However, further experiments on larger and more diverse groups of subjects are necessary to validate these biomechanical tools and subsequently adopt them at a larger scale. Furthermore, it is important to keep in mind that the analysis of movements and posture alone is not informative enough and should be combined with additional measures [29, 32, 35, 36, 42].

Clinical strategies applied to forensic practice involve qualitative evaluations of symptoms and behaviours based on clinical and epidemiological criteria. For example, they target patterns of cognitive and/or psychological symptoms that are unlikely, amplified, excessive, or unexpected. These detection strategies, such as SVT or the *floor effect* strategy, are widely used for forensic purposes and are the basis for numerous questionnaires and instruments [44, 45]. Nevertheless, experimental applications in the context of WAD are still scarce. Among these clinical instruments, the Sobel test [50] and other related simulation tests [55] that require the presence of a professional to be administered focus on the evaluation of non-organic symptoms to identify abnormal illness behaviours. However, they still lack evidence-based validation and currently present several areas of conflicting evidence in the literature [53, 54]. Numerous neuropsychological tests have been developed to detect malingering in forensic contexts, especially regarding the assessment of memory deficits (e.g., RWRL, TOMM, ASTM). However, evidence for the efficacy of these tools, specifically in the context of WAD, is lacking. Instead, various self-report questionnaires have been designed or adapted for the detection of malingering among patients manifesting whiplash-related symptoms (e.g., IPQ-R, MMPI-CCS, SIMS and SRSI), though future research is recommended to establish shared cut-off scores, assess the quality of the measures and their reliability, and validate them in larger groups. The SCL-90-R [62] and the WSQ [26] appear particularly promising, as they are able to identify characteristic profiles of those who have suffered whiplash-related injuries that are difficult to replicate by dishonest claimants. Notably, despite the widespread use of self-reported measures, they share an important limitation: they rely on patients’ reports of their symptoms and are potentially vulnerable to coaching [84]. Given the variability and subjectivity that characterise WADs, this may represent a serious issue [16]. Moreover, the literature suggests that, in contrast to English-speaking countries, there is a remarkable lack of adequate tools to examine symptom simulation in non-Anglophone countries [73]. This may represent an important point, given the various country-level rates of whiplash-related injury claimants [2]; thus, self-reported questionnaires need to be validated, linguistically and culturally, for use in other countries to allow a realistic representation of the presence of malingering in whiplash-related injury reports*.*

Finally, cognitive-based lie detection techniques represent a particularly innovative field that opens a range of opportunities to provide objectivity in the evaluation of WAD and in the detection of malingering. To date, only the aIAT has been tested to identify malingered presentations in the context of whiplash [83]. The findings are encouraging; however, further validations are necessary. Moreover, there is increasing evidence to suggest that kinematic analyses combined with other cognitive load-inducing techniques, such as complex sentences [77] or unexpected questions [78], can be used as an implicit measure of the real-time mental processes involved in decision-making [85], thus representing valid alternatives for detecting malingered symptom presentations [45]. For example, the analysis of mouse movements in a forced-choice computerized task was effectively applied to detect the simulation of depression [16] and amnesia [79]. However, the literature on this front, albeit rapidly expanding, is still in its infancy, and further research is needed to test and validate these strategies for the detection of malingered WADs. Similarly, the application of artificial intelligence (e.g., machine learning and neural networks) models to classify data represents an interesting area for future investigations [86].

Overall, a significant number of the studies reviewed are dated, lack replication, tested small sample sizes, adopted non-controlled and heterogeneous designs, or did not assess the quality of the instruments (e.g., specificity or accuracy indexes, inter-rater reliability) used. Many works have inconsistent findings, which may be due to several reasons. First, different studies focused on different categories of symptoms, such as cognitive impairments, biomechanical signs, or psychological disturbances. Importantly, no single test can perfectly identify malingered symptom presentations and claims, and a range of instruments is necessary for accurate evaluations to detect dishonest individuals [32]. Secondly, there are inconsistencies among the designs of the studies (e.g., within- vs. between-subjects vs. mixed designs, experimental instructions, malingering indexes considered) [48]. For instance, different cut-off scores were used for the same measures, and different methods were adopted to assess malingering and define it [48]. Furthermore, a variety of populations were tested, ranging from healthy subjects asked to feign their symptoms to groups of real-life claimants. Notably, the pattern of performance of instructed malingerers may not be comparable to those performed by true malingerers [29, 35, 48]. Moreover, control groups are not always included and, where present, are largely heterogeneous. Altogether, these elements make the comparison between studies and malingering detection strategies problematic.

## Conclusions

The present review provides an up-to-date overview of the strategies that have been used to detect malingered symptoms following whiplash and raises awareness of the urgent need for controlled, high-quality studies in this field that can aid in the development of evidence-based guidelines and validated protocols for the assessment of symptoms following whiplash-related injuries. Despite the advances made and the promising new avenues offered by recent technological developments, the present work highlights the current lack of standardised procedures and evidence-based guidelines for the assessment of whiplash-related symptoms that consider both the heterogeneous nature of the syndrome and the possibility of malingering. Keeping in mind that no single test can detect dishonest compensation claims and that a multi-dimensional evaluation is necessary [32], high-quality controlled studies in the field of WAD and malingering detection in WAD that can aid in the development of shared evaluation procedures are strongly encouraged. Novel studies need to include larger sample sizes, control groups, systematic and accurate analyses of the data, and a quality assessment of the measures tested. Moreover, when whiplash symptoms are present, a thorough and exhaustive evaluation should be performed, combining (a) one or more non-invasive biomechanical tools that allow the assessment of motor impairments and (b) clinical tools suitable for forensic applications that are able to identify the presence of rare, impossible, or exaggerated patterns of symptoms. These tools should be chosen according to the symptoms reported by each patient, such as memory problems and cognitive impairment or psychological disturbances. Moreover, the recent technological developments in the field of lie detection have led to promising, cost-effective, and easy-to-administer techniques (e.g., aIAT, mouse dynamics, and keystroke dynamics analysis) that may be particularly beneficial for the detection of malingered WADs, especially when coupled with artificial intelligence algorithms, and should be included in the evaluation procedure.

On a final note, it is important to remember that, even though malingering may be prominent among whiplash cases and its eventuality should be always considered, genuine WADs also exist and cannot be neglected. Considering this, the benefits of a comprehensive, careful, and standardized assessment that allows accurate differential diagnoses are evident.

## Data Availability

Not applicable.
